# Hydraulic forces assist left ventricular filling in aortic stenosis at baseline and after valve replacement

**DOI:** 10.1016/j.jocmr.2026.102722

**Published:** 2026-04-03

**Authors:** Bryce Watson, Jonathan Bennett, Nikoo Aziminia, Abhishek Shetye, George D. Thornton, Rhodri Davies, Iain Pierce, Rebecca Kozor, Alun D. Hughes, James C. Moon, Martin Ugander, Thomas A. Treibel

**Affiliations:** a Institute of Cardiovascular Science, University College London, London, United Kingdom; b St George Physician Training Network, Sydney, Australia; c Department of Cardiovascular Imaging, Barts Heart Centre, St Bartholomew’s Hospital, London, United Kingdom; d Kolling Institute, Department of Cardiology, Royal North Shore Hospital, and University of Sydney, Sydney, Australia; e Department of Clinical Physiology, Karolinska University Hospital, and Karolinska Institutet, Stockholm, Sweden

**Keywords:** Aortic stenosis, Remodelling, Diastology

## Abstract

**Background:**

Diastolic dysfunction in the setting of aortic valve replacement (AVR) for aortic stenosis (AS) is incompletely understood. This study aims to assess the net hydraulic force of left ventricular (LV) filling in participants with severe symptomatic AS undergoing AVR.

**Methods:**

This single-centre prospective observational cohort study evaluated patients with severe, symptomatic AS undergoing AVR between 2012 and 2015. Clinical assessment and cardiovascular magnetic resonance were completed prior to AVR and 1-year post-operatively. Atrioventricular area difference (AVAD) was used as a surrogate for the hydraulic force of LV filling. AVAD at mid-diastole was measured as the difference between LV short-axis area and left atrial short-axis area.

**Results:**

In patients with AS (n = 110, 54% [59/110] male, age 71 [64–77] years, aortic valve area 0.74 ± 0.25 cm^2^), AVAD was positive at baseline (2.8 ± 6.5 cm^2^), consistent with a net hydraulic force assisting LV filling. While AVAD did not change post-operatively on a group level (p = 0.70), an improvement in AVAD was associated univariably with increasing baseline LV ejection fraction, and decreasing baseline AVAD, LV volume, mass, myocardial extracellular volume, and infarct size (p<0.05 for all), and multivariably with baseline decreasing AVAD, LV mass, and age (model adjusted R^2^ = 0.49, p<0.001).

**Conclusion:**

In severe AS, hydraulic force contributes to LV filling prior to and following AVR. The greatest improvement in hydraulic force following AVR occurred in those with the lowest baseline hydraulic force, but also with lower age and the absence of otherwise deleterious LV myocardial remodelling.

## Graphical abstract

**Figure:**
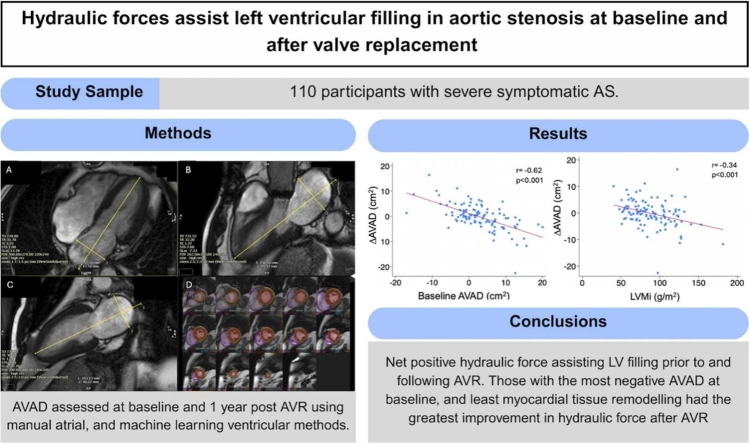

## 1 Introduction

Aortic stenosis (AS) is the most common valvular heart disease (VHD) in developed countries and is becoming increasingly more prevalent due to aging populations [1]. Given the prevalence in elderly individuals, definitive treatment of AS with either open surgical or transcatheter aortic valve replacement (AVR) [2] merits careful consideration. Half of all individuals with AS, and two-thirds of those having AVR for severe AS have diastolic dysfunction [3], [4]. Impaired diastolic function pre-operatively has been demonstrated to be a predictor of poor outcomes including mortality following AVR [3], [4], [5]. A meta-analysis has shown higher all-cause mortality and major adverse cardiovascular events following transcatheter AVR in those with severe diastolic dysfunction [6]. This relationship appears to be dependent on severity, with those stratified into higher grades of diastolic dysfunction pre-operatively having increased rates of cardiovascular death or hospitalization following AVR [7].

Reverse remodelling has been demonstrated following AVR with reduction in left ventricular (LV) mass and regression of diffuse myocardial fibrosis seen in the first year following intervention [8], [9], [10], [11]. However, this reverse remodelling does not necessarily lead to improvement in diastolic function [12]. The echocardiographic grade of diastolic dysfunction pre-operatively has been shown to improve following AVR in between 50%–71% of cases [13], [14]. Those whose grade of diastolic dysfunction improves following AVR have lower rates of death and hospitalisation at 1 and 2 years post-intervention [13]. This lack of improvement in diastolic function in some individuals despite improvements in myocardial hypertrophy and diffuse myocardial fibrosis is incompletely understood and warrants further investigation.

The forces that contribute to diastolic LV filling include active relaxation [15], elastic restorative forces [16], left atrial contraction [17], and hydraulic force [18]. The hydraulic force of LV filling is determined by the geometric relationship between the LA and LV [18]. The impact of this force on the AV plane (mitral valve annulus and associated structures) is of particular importance as it influences AV plane motion during diastole. When the mitral valve is open the pressure is approximately equal between the LA and LV. Due to Pascal’s principle (Pressure = Force/Area), the forces acting on the AV plane are determined by the LA and LV short-axis areas. The physical model of a hollow piston has been used to demonstrate how a difference in atrial and ventricular short-axis surface areas causes the AV plane to move in an apex-to-base direction [18], assisting LV filling. The atrioventricular area difference (AVAD) is a measure of the difference between the ventricular short-axis area (VSA) and the atrial short-axis area (ASA). AVAD can therefore be used as a surrogate for the hydraulic force acting on the AV plane. The hydraulic force is estimated to contribute 10%–60% of the driving force of LV filling [18] and is an important aspect of diastolic function that warrants further investigation.

This force is yet to be assessed in individuals with AS or in those having AVR. It is not known whether the remodelling and reverse remodelling seen in AS will impact the hydraulic force. Hydraulic forces have been shown to provide independent prognostic information beyond diastolic dysfunction grading [19] and may provide insights into diastolic dysfunction in AS, particularly persisting diastolic dysfunction following AVR. This study aims to analyze the atrioventricular area difference (AVAD) as a surrogate for the hydraulic force of LV filling in participants with severe AS before and after AVR and investigates its relationship with known markers of myocardial function and tissue remodelling. Previous studies have investigated the hydraulic force throughout the cardiac cycle in a small number of participants [18,^24^,25]. Elastic restorative forces and active relaxation occur in early diastole, and atrial contraction occurs in late diastole, therefore, this study focuses on the hydraulic force in mid-diastole where it has the greatest proportional impact on LV filling.

## 2 Methods

This is a sub-study of the prospective RELIEF-AS study of patients who had AVR for severe AS between January 2012 and January 2015 (NCT02174471) [8]. The participants were from a single referral centre (University College London Hospitals NHS Trust, London) and were recruited prior to having their intervention. This study was approved by the United Kingdom National Research Ethics Service (19/LO/1849). Written informed consent was obtained from all participants and the study conformed to the principles of the Declaration of Helsinki. The participants completed clinical, biochemical, 6-minute walk test (6MWT) [20], echocardiographic, and cardiovascular magnetic resonance (CMR) assessments at baseline and 1-year following AVR.

### 2.1 Study sample

Participants were individuals with severe symptomatic AS who underwent AVR with or without coronary artery bypass graft (CABG). Those over the age of 18 with severe AS were included. Severe AS was defined as two or more of the following echocardiographic parameters: aortic valve area (AVA) <1 cm^2^, velocity time integral ratio <0.25, aortic valve peak pressure gradient ≥64 mmHg, or mean pressure gradient ≥40 mmHg. Those with severely impaired renal function (estimated glomerular filtration rate <30 mL/min/1.73 m^2^), previous cardiac valve procedures, infective endocarditis, severe mitral regurgitation (MR), severe aortic regurgitation (AR), pseudo-severe AS, cardiac amyloid, Fabry disease, insufficient CMR acquisitions for AVAD analysis, those pregnant or breast feeding, or those whose severe AS was managed with medical therapy alone were excluded from the study.

### 2.2 Cardiovascular magnetic resonance

CMR was performed using a standard clinical protocol at 1.5 Tesla (Magnetom Avanto, Siemens Healthineers, Erlangen, Germany). Late gadolinium enhancement (LGE), and T1 mapping by Modified Look-Locker Inversion recovery were performed before and after administration of gadolinium contrast (gadolinium-DOTA, Guerbet S.A., Villepinte, France). LGE imaging was completed 10 min post contrast administration and analysed as regions with a signal >3 SD from remote. T1 mapping was performed before and 15 min post contrast for calculating myocardial extracellular volume fraction (ECV). Images were analysed on CVI42 software version 5.12 (Circle Cardiovascular Imaging, Calgary, Alberta, Canada). Global thickness (GT) was calculated using the equation: GT = 0.05 + (1.60 x LVM[*g*]^0.84^ x LVEDV[ml]^−0.49^) [21].

### 2.3 Atrioventricular area difference analysis

Endocardial ventricular short-axis area (VSA) measurement was performed with the assistance of a validated machine learning model [22]. The model identified the endocardial contours to measure the VSA and was manually corrected as needed. Papillary muscles were included in the blood volume, and CMR slices including the left ventricular outflow tract were excluded.

**Fig. 1:**
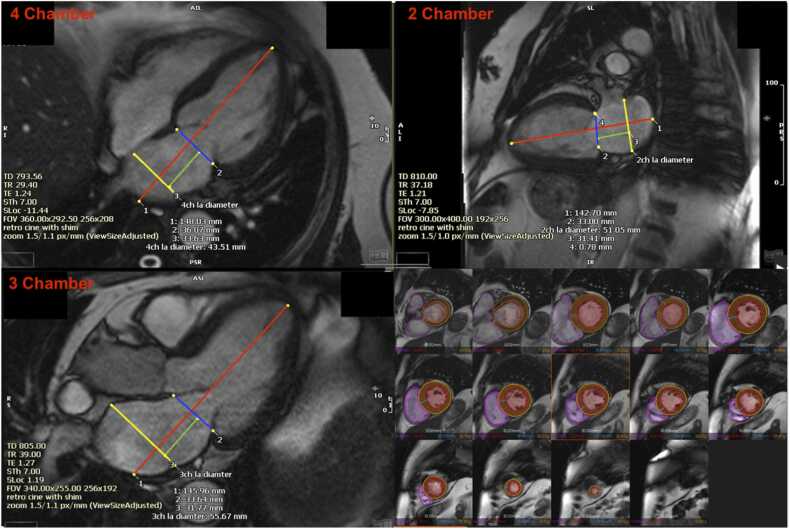
ASA and VSA Methodology. ASA Methodology: 4 chamber, 3 chamber, and 2 chamber long axis acquisitions, all taken at mid-diastole. The long axis of the LV is marked (red). The location of atrioventricular plane is marked (blue). The maximal LA diameter perpendicular to the LV long axis is measured in yellow. The mean of these three LA diameter measurements was used to assess ASA using circular approximation. Distance from mitral valve annulus to the maximal LA diameter on each acquisition is measured in green to ensure all are within 5 mm of each other. VSA Methodology in lower right panel: Short-axis acquisitions through the LV at mid-diastole. VSA measured with validated machine learning model in each acquisition. Maximum VSA was used to calculate AVAD. *LV* left ventricle, *LA* left atrium, *ASA* atrial short-axis area, *VSA* ventricular short-axis area, *AVAD* atrioventricular area difference

Atrial short-axis area (ASA) was assessed using a circular approximation method from the mean of 3 manually measured maximum LA diameters. The short-axis acquisitions could not be used to measure the ASA as they incompletely imaged the base of the LA and therefore may not have included the maximum ASA. Diameter measurements were obtained on 4-chamber, 3-chamber and 2-chamber long axis acquisitions. The maximum short-axis LA diameters on each acquisition were measured perpendicular to the long axis of the left ventricle (Fig. 1) and excluded the atrial appendage. All measurements were made at the same distance from the mitral valve annulus within 5 mm of each other. The ASA was calculated from the mean of the maximum atrial short-axis diameters using circular approximation from the radius of the short-axis measurements.

AVAD was used as a surrogate measurement for the net hydraulic force acting on the AV plane, and was measured on CMR at mid-diastole as follows:

AVAD [cm^2^] = VSA [cm^2^] – ASA [cm^2^]

Change in AVAD (ΔAVAD) was calculated as post-operative AVAD minus pre-operative AVAD.

### 2.4 Echocardiography

Transthoracic echocardiography was performed using Vivid E9 system (GE Healthcare, Waukesha, Wisconsin, ) and measurements were performed according to the guidelines of the American Society of Echocardiography and European Society of Echocardiography [23].

### 2.5 Biochemistry

Enzyme Linked Immunosorbent Assay was used to analyse serum N-terminal pro-brain natriuretic peptide (NT-proBNP) and high-sensitivity troponin T (hsTnT) (Roche Diagnostics, Indianapolis, Indiana, USA).

### 2.6 Statistical analysis

Statistical analysis was performed on STATA-MP Version 18.0 (StataCorp, College Station, Texas, USA). Normality was assessed with quantile-quantile plots and Shapiro-Wilk tests. Unpaired t-tests were used to compare means between two groups of normally distributed continuous variables and paired t-tests if these variables were paired. Pearson correlation coefficients were used to assess linear correlations between two continuous variables and Spearman non-parametric rank correlation coefficients for monotonic non-linear relationships between continuous variables. Multivariable linear regression was used to adjust correlations for potential confounders. Confounders were chosen based on prior evidence that they might be common causes of the exposure and outcome. Highly skewed variables were log-transformed prior to inclusion in models. All analyses were based on complete case data. Missing data were addressed using listwise deletion.

## 3 Results

### 3.1 Baseline study population

**Fig. 2:**
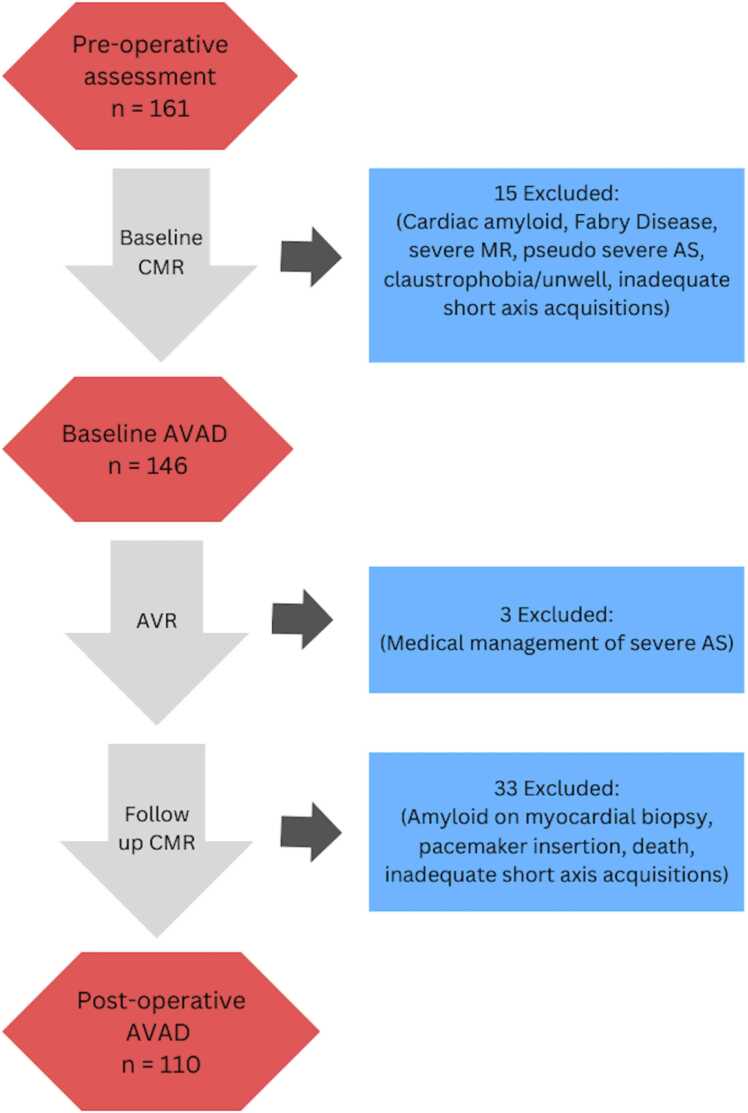
Study consort diagram. *CMR* cardiovascular magnetic resonance, *AS* aortic stenosis, *MR* mitral regurgitation, *AVAD* atrioventricular area difference, *AVR* aortic valve replacement

161 participants completed pre-operative assessment including CMR. Fifty-one were excluded from the study prior to analysis (Fig. 2). The median age of the 110 included patients was 71 years and 54% (59/110) were male. LVEF was 70 [64–80] %, peak aortic valve velocity 4.4 ± 0.6 m/s, and mean aortic valve gradient 47 ±14 mmHg (Table 1).

**Table 1:** Baseline participant characteristics

Baseline characteristics	n = 110
*Clinical*	
Male sex, n (%)	59 (54)
Age, years	71 [64–77]
BMI preop, kg/m^2^	28.8±4.9
BSA, m^2^	1.89±0.2
Diabetes, n (%)	21 (19)
Hypertension, n (%)	83 (75)
Coronary Artery Disease, n (%)	32 (29)
NYHA class I, II, III, IV, n (%)	14 (13), 53 (50), 37 (35), 2 (2)
Preop 6MWT, m	471±185
*Echocardiography*	
AVA index, cm^2^/m^2^	0.39±0.13
Peak AV velocity, m/s	4.4±0.6
Mean Gradient, mmHg	47±14
Peak Gradient, mmHg	77±20
e' mean, cm/s	6.5±2.1
E/e' average, unitless	13.3 [9.1–16.0]
PASP, mmHg	31±8
*CMR Parameters*	
LVEF, %	70 [64–80]
LVEDVi, ml/m^2^	66±23
LVMi, g/m^2^	88±26
ECVF, %	28.1±2.8
Global thickness, mm	11.4±2.1
LGE, g	9[5–17]
*Biomarkers*	
hsTNT, pmol/L	14[9–20]
NT-proBNP, ng/L	71 [30–225]

Participants characteristics of those that completed baseline AVAD.

Values are numbers (%) of cases, mean ± standard deviation, or median [interquartile range]. *BMI* body mass index, *NYHA* New York heart Association, *6MWT* 6 min walk test, *AVA* aortic valve area, *AV* aortic valve, *e’* mean velocity of mitral valve annulus displacement, *E* peak early velocity of transmitral flow, *PASP* pulmonary artery systolic pressure, *CMR* cardiac magnetic resonance imaging, *LVEF* left ventricular ejection fraction, *LVEDVi* left ventricular end diastolic volume indexed to body surface area, *LVMI* left ventricular mass index, *ECVF* extracellular volume fraction of the left ventricle, *LGE* late gadolinium enhancement using 3 SD method, *hsTNT* high-sensitivity troponin T, *NT-proBNP* N-terminal pro-brain natriuretic peptide

### 3.2 Baseline AVAD

Baseline AVAD was 2.8 ± 6.5 cm^2^ consistent with a net positive hydraulic force assisting LV filling. AVAD trended towards being more positive in males compared to females (males 3.8 ± 7.0 cm^2^ vs females 1.7 ± 5.7 cm^2^, p = 0.09). Baseline AVAD was negatively correlated with age (rho = −0.40, p<0.001).

### 3.3 Baseline AVAD correlations

Correlations between baseline AVAD and baseline clinical, biochemical, echocardiographic, and CMR variables are summarized in Table 2. The strongest correlations were between baseline AVAD and baseline LV end-diastolic volume index (LVEDVi) (r = 0.58, p<0.001), LVEF (r = −0.48, p<0.001), and LV mass index (LVMi) (r = 0.46, p<0.001). Correlations were also present between baseline AVAD and age, diffuse myocardial fibrosis (ECV), and focal myocardial fibrosis (LGE).

**Table 2:** Baseline AVAD correlations with baseline variables

Baseline Variable	Correlation (r)	Comparison of means	p-value
Age	**−0.39**^†^	-	**<0.001**
Sex	-	Males: 3.8±7.0 cm^2^ Females: 1.7±5.7 cm^2^	0.09
Coronary Artery Disease	-	Present 3.4±8.1 cm^2^ Absent 2.5±5.8 cm^2^	0.51
Hypertension	-	Present 2.4±6.6 cm^2^ Absent 4.0±6.1 cm^2^	0.28
Baseline 6MWT	0.19	-	0.07
ln(hsTNT)	**0.22**	**-**	**0.02**
ln(NT-proBNP)	**0.25**	**-**	**0.01**
e'	−0.06	-	0.58
E/e'	**0.18**	-	**0.09**
LVEF	**−0.48**	**-**	**<0.001**
LVEDVi	**0.58**	**-**	**<0.001**
LVMi	**0.46**	**-**	**<0.001**
Ln LGE	**0.22**	**-**	**0.02**
ECV	**0.34**	**-**	**<0.001**

Correlations between baseline Atrioventricular Area Difference and baseline clinical, biochemical, and imaging variables, n =110.

Values are means ± standard deviation.

Pearson’s correlation is used for parametric data and Spearman’s Rank correlation (†) for non-parametric data. *6MWT* 6 min walk test, *hsTNT* high-sensitivity troponin T, *NT-proBNP* N-terminal pro-brain natriuretic peptide, *e’* mean velocity of mitral valve annulus displacement, *E* peak early velocity of transmitral flow, *LVEF* left ventricular ejection fraction, *LVEDVi* left ventricular end diastolic volume indexed to body surface area, *LVMi* left ventricular mass index, *LGE 3 SD* late gadolinium enhancement using 3 SD method, *ECV Fraction* extracellular volume fraction of the left ventricle

### 3.4 Intervention

Five participants had TAVI (5%), 68 had tissue SAVR (62%), 33 had mechanical SAVR (30%), and 4 had suture-less SAVR (4%). Thirty-five participants (32%) had concomitant coronary artery bypass grafting (CABG).

### 3.5 Associations with changes in AVAD

Correlations between change in AVAD (ΔAVAD) and baseline clinical, biochemical, echocardiographic, and CMR variables were assessed with key findings summarized in Table 3. In univariable analysis, there were negative correlations between ΔAVAD and ECV, LVMi, LVEDVi, NT-pro-BNP, and hsTNT. Lower baseline values of these variables were associated with a larger increase in AVAD after AVR. The strongest correlation was a negative correlation between ΔAVAD and baseline AVAD. In a multivariable model adjusting for potential confounders, baseline AVAD (standardized beta = −0.65, p<0.001), age, and LVMi were independently associated with ΔAVAD. The overall multivariable model adjusted R^2^=0.49 (p<0.001). When comparing ΔAVAD between those whose baseline AVAD was below or above the median of baseline AVAD (2.4 cm^2^), those below the median had a greater ΔAVAD (2.1 ± 4.0 vs −2.5 ± 5.0 cm^2^, p<0.001). Baseline and post-operative AVAD did not differ overall (2.8 ± 6.5 vs 2.6 ± 5.2 cm^2^, p = 0.70).

**Table 3:** Correlations between ΔAVAD and baseline variables

Baseline variable	Correlation coefficient	Difference in means	Univariable p-value	Standardized Multivariable coefficient	Multivariable p-value
Sex		+0.67cm^2^ (Males: −0.50 +/- 4.80 cm^2^ Females:0.17 +/- 5.30 cm^2^)	0.485	0.07	0.416
Age, years	0.01	-	0.887	**−0.26**	**0.003**
6MWT, meters	0.05	-	0.621	-	-
ln(hsTNT), ln(ng/ml)	**−0.31**	**-**	**0.001**	**-**	**-**
ln(NT-proBNP), ln(pq/ml)	**−0.33**	**-**	**<0.001**	**-**	**-**
e', cm/s	0.06	-	0.594	-	-
E/e', unitless	−0.20	-	0.072	-	-
LVEF, %	**0.32**	**-**	**<0.001**	0.01	0.923
LVEDVi, ml/m^2^	**−0.31**	**-**	**0.001**	0.25	0.053
LVMi, g/m^2^	**−0.34**	**-**	**<0.001**	**−0.28**	**0.021**
ln(LGE), ln(g)	**−0.19**	-	**0.045**	0.02	0.057
ECV, % LV	**−0.35**	**-**	**<0.001**	−0.15	0.071
Baseline AVAD, cm^2^	**−0.62**		**<0.001**	**−0.65**	**<0.001**
				**Multivariable model adjusted R**^**2**^**= 0.49, p<0.001**	

Correlations between change in AVAD (ΔAVAD) and baseline clinical, biochemical, and imaging variables.

Values are means ± standard deviation.

Multivariable model included sex, age, LVEF, LVEDVi, LVMi, Ln(LGE), and ECV.

*6MWT* 6 min walk test, *hsTNT* high-sensitivity troponin T; *NT-proBNP* N-terminal pro-brain

natriuretic peptide, *LVEF* left ventricular ejection fraction, *LVEDVi* left ventricular end diastolic volume indexed to body surface area, *e’* mean velocity of mitral valve annulus displacement, *E* peak early velocity of transmitral flow, *LVMi* left ventricular mass index; *LGE* late gadolinium enhancement using 3 SD method, *ECV Fraction* extracellular volume fraction of the left ventricular myocardium

### 3.6 Echocardiographic diastolic function

Echocardiographic diastolic function improved following AVR. E/e’ decreased (12.5 [9.1–16.0] vs 10.3 [7.8–12.6], p<0.01) and e’ increased (6.6 ± 2.1 vs 8.1 ± 2.7 cm/s, p<0.01). No correlation was found between baseline AVAD or ΔAVAD and baseline E/e’ or e’ velocity (p>0.05 for all).

**Fig. 3:**
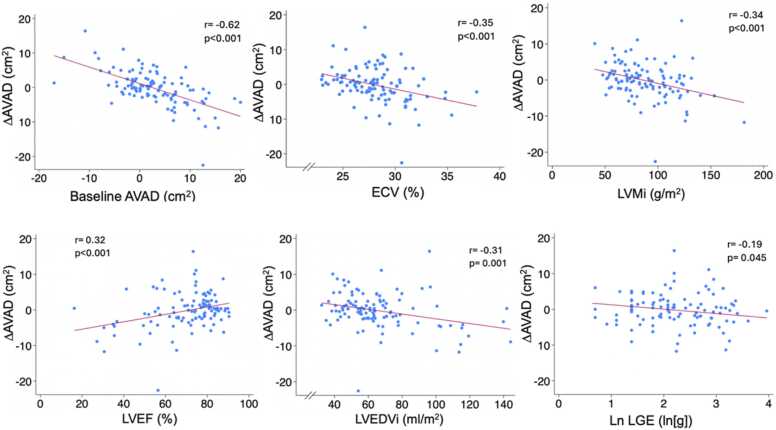
ΔAVAD correlations with baseline parameters. *ΔAVAD* change in atrioventricular area difference (cm^2^), *ECV* left ventricular extracellular volume fraction (%), *LVMi* left ventricular mass index (g/m^2^), *LVEF* left ventricular ejection fraction (%), *LVEDVi* left ventricular end diastolic volume (ml/m^2^), *LGE* late gadolinium enhancement (g)

### 3.7 Functional outcome

No convincing correlation was found between baseline 6MWT and either baseline AVAD (r = 0.19, p = 0.07) or ΔAVAD (r = 0.05, p = 0.62). However, post-operative AVAD was correlated with post-operative 6MWT (r = 0.39, p<0.01).

## 4 Discussion

This was the first study assessing the hydraulic force of LV filling in AS and following AVR. The study demonstrated that AVAD, as a surrogate for the hydraulic force, was positive at baseline and post-operatively, therefore assisting LV filling in both instances. While AVAD did not change overall following AVR, improvement in AVAD associated inversely with baseline AVAD, and this association persisted in multivariable analysis. Notably, improvement in AVAD associated with lower age and the absence of otherwise deleterious LV myocardial remodeling (increased LVMI & ECV), possibly supporting the benefits of early intervention.

A positive AVAD at baseline and following AVR is consistent with a net hydraulic force acting on the AV plane in an apex-to-base direction which assists LV filling in AS. This physiological process occurs in addition to active relaxation, elastic restorative forces, and left atrial contraction. Previous studies have also found a net positive hydraulic force in pathologies such as heart failure with reduced ejection fraction [24] and atrial septal defects (ASDs) [25].

On average, in this study, the AVAD did not change following AVR. This contrasts with studies investigating AVAD in other pathologies and interventions. In a study assessing the hydraulic force before and after closure of atrial septal defects an increase in AVADi (AVAD indexed to body surface area) from a median of 6.4 cm/m^2^ to 8.7 cm/m^2^ was observed [25]. ASD closure changes the volume loading between the heart chambers and therefore impacts left atrial and left ventricular size and consequently AVAD. Changes in volume loading, such as due to atrial septal defects, may cause more dynamic changes in chamber size than interventions for pressure loading conditions like AS [26], [27]. This is a potential explanation for why the mean hydraulic force did not change discernibly in this study.

In contrast, pressure loading pathologies have been shown to cause a greater degree of LA enlargement than volume loading pathologies [27]. This knowledge inspired the hypothesis of the current study, namely that AVAD would increase post-AVR, due to a decreased LA size after intervention. However, this study did not demonstrate a decrease in ASA or LA size following AVR. A 2024 meta-analysis of 1066 patients assessing LA size following TAVI demonstrated only a modest 2.7 mL/m^2^ reduction in LA volume indexed to body surface area (LAVi) [28]. This is only a 5% reduction in volume from baseline and such a small change may not have been detectable in our study. In general, there is a paucity of research investigating how the hydraulic force changes with different valvular pathologies and interventions.

A negative correlation between ΔAVAD and baseline AVAD was observed in this study. These results suggest that those with the lowest baseline AVAD have the greatest improvement in hydraulic force following AVR. Regression towards the mean is a potential alternative explanation for this as there was no change between baseline and post-operative AVAD. However, this regression can occur even in the absence of the above-observed ΔAVAD and baseline AVAD correlation.

Following removal of the increased afterload with AVR, reverse myocardial tissue remodelling takes place within the first year [9]. Prior research has demonstrated that post-AVR, there is a more pronounced regression in LVMi and diffuse myocardial fibrosis in those with the highest values of these parameters at baseline [29], [30]. However, in these studies, higher LVMi at baseline was not shown to correlate with geometric reverse remodelling following AVR [29].

A more recent publication has hypothesized that a lack of geometric improvement seen in some individuals post-AVR is attributable to the presence of myocardial fibrosis [31]. The findings of the present study indicate that the most marked improvement in AVAD post AVR occurs in those with the poorest baseline AVAD, but the least severe myocardial tissue remodelling phenotype (lower LVMi, ECV), supporting this hypothesis. Further research is needed to determine whether early intervention in AS before marked myocardial tissue remodelling occurs allows for greater improvements in hydraulic force post AVR.

Baseline AVAD did not associate with baseline 6MWT, likely related to the fact that all subjects had severe AS at baseline. However, post-operative AVAD was positively associated with post-operative functional capacity. Therefore, those with the best hydraulic force following AVR also had the best functional outcome.

## 5 Limitations

Circular approximation was used to calculate the ASA, as opposed to measuring the true area, which will limit the accuracy of the ASA component used for the AVAD. Ideally, ASA would be measured using the short-axis acquisitions using the same machine learning model as VSA. However, the CMR protocol used to acquire short-axis images did not fully cover the left atria and therefore could not be used to assess the maximum ASA. AVAD was measured during mid-diastole in this study. The hydraulic force of LV filling acts throughout the cardiac cycle and changes as the geometric relationship between the left atrium and left ventricles changes. Previous studies have measured the net hydraulic force throughout the cardiac cycle to demonstrate it in a small number of participants [18]. Mid-diastole was deemed to be the most useful single timepoint to measure the hydraulic force, as it is when it has the greatest proportional impact on LV filling. This is in light of active relaxation and elastic restorative forces occurring in early diastole, and atrial contraction in late diastole. This study focuses on the hydraulic force as a single component of diastolic function because its impact has not previously been investigated in AS or AVR. Many other important aspects of diastolic function, such as active relaxation, atrial contraction, and flow dynamics are not specifically assessed in this study. AVAD is used as a surrogate marker for the hydraulic force acting on the AV plane opposed to measuring the true hydraulic force as this would require invasive pressure measurements.

As only participants with severe symptomatic AS were included, the results of this study may not be applicable to those with asymptomatic AS, or less advanced stages of AS.

## 6 Conclusions

This is the first study assessing the hydraulic force of LV filling in a cohort of participants with severe aortic stenosis undergoing AVR. There was a net positive hydraulic force assisting left ventricular filling prior to and following valve replacement. Those with the most negative AVAD at baseline have the greatest improvement in hydraulic force following intervention. However, this improvement in hydraulic force is most marked in those yet to develop severe myocardial tissue remodelling.

## Funding

The original funding for the RELIEF-AS study was supported by doctoral research fellowships by the National Institute of Health Research (NIHR; DRF-2013–06-102) and British Heart Foundation (FS/19/35/34374), respectively. This project was funded by the European Commission FP7 Programme, Brussels, Belgium (FIBRO-TARGETS project 2013–602904).

## Author contributions

**Bryce Watson:** Writing – review & editing, Writing – original draft, Visualization, Validation, Methodology, Investigation, Formal analysis, Data curation, Conceptualization. **Jonathan Bennett:** Writing – review & editing, Validation, Supervision, Methodology, Investigation, Conceptualization. **Nikoo Aziminia:** Writing – review & editing, Supervision, Methodology. **Abhishek Shetye:** Writing – review & editing. **George D. Thornton:** Writing – review & editing, Supervision. **Rhodri Davies:** Writing – review & editing, Software, Methodology, Investigation, Formal analysis, Conceptualization. **Iain Pierce:** Writing – review & editing, Software, Data curation. **Rebecca Kozor:** Writing – review & editing, Investigation. **Alun D. Hughes:** Writing – review & editing, Conceptualization. **James C. Moon:** Writing – review & editing. **Martin Ugander:** Writing – review & editing, Visualization, Validation, Supervision, Methodology, Investigation, Formal analysis, Conceptualization. **Thomas A. Treibel:** Writing – review & editing, Supervision, Resources, Project administration, Methodology, Funding acquisition, Formal analysis, Conceptualization.

## Availability of data and materials

The data for this study is available from the corresponding author upon reasonable request.

## Declaration of Competing Interest

The authors declare the following financial interests/personal relationships which may be considered as potential competing interests. T.A. Treibel reports financial support was provided by National Institute for Health and Care Research. T.A. Treibel reports financial support was provided by British Heart Foundation. T.A. Treibel reports financial support was provided by European Commission. If there are other authors, they declare that they have no known competing financial interests or personal relationships that could have appeared to influence the work reported in this paper.
